# Biogenic Synthesis of Silver-Core Selenium-Shell Nanoparticles Using *Ocimum tenuiflorum* L.: Response Surface Methodology-Based Optimization and Biological Activity

**DOI:** 10.3390/nano11102516

**Published:** 2021-09-27

**Authors:** Femi Olawale, Mario Ariatti, Moganavelli Singh

**Affiliations:** Nano-Gene and Drug Delivery Group, Discipline of Biochemistry, School of Life Sciences, College of Agriculture, Engineering and Science, University of KwaZulu-Natal, Private Bag X54001, Durban 4000, South Africa; 219094535@stu.ukzn.ac.za (F.O.); ariattim@ukzn.ac.za (M.A.)

**Keywords:** silver, selenium, bimetallic nanoparticles, green synthesis, response surface methodology, antioxidant, cytotoxicity, *Allium cepa* assay

## Abstract

Bimetallic nanoparticles (BNPs) have shown better biological potential compared to their monometallic counterparts owing to the synergistic effect produced by these alloys. In this study, selenium-capped silver nanoparticles (Ag@Se NPs) were synthesized using an *Ocimum tenuiflorum* extract. These BNPs were characterized using UV-visible, Fourier transform infrared spectroscopy, nanoparticle tracking analysis, electron microscopy and energy dispersive x-ray analysis. Response surface methodology was used to understand how extract volume and temperature influenced the zeta potential, hydrodynamic size and NP concentration. The phytoconstituents were identified using gas chromatography-mass spectrometry (GC-MS) and molecular docking studies were performed on B-DNA to determine possible genotoxicity. Antioxidant activities, in vitro cytotoxicity (3-(4,5-dimethyl thiazol-2-yl)-2,5-diphenyl tetrazolium bromide (MTT) assay), and genotoxicity (*Allium cepa* root cells) of these BNPs, were also evaluated. A surface plasmon resonance band around 420 nm confirmed BNP formation with significant quantities of silver and selenium. The Ag@Se NPs displayed good stability, dispersity, antioxidant activity, and compatibility at low concentrations but showed significant cytotoxicity and genotoxicity at high concentrations. Molecular docking analysis showed weak interactions between the plant constituents and B-DNA, suggesting no genotoxicity. These results provide an insight into the conditions required for optimal production of eco-friendly Ag@Se NPs with interesting biological properties.

## 1. Introduction

The area of inorganic nanoparticle (NP) synthesis has grown over the years to include nano-sized alloys, which may contain two or more inorganic materials. The formulations of bimetallic nanoparticles (BNPs) are by far the most studied and have been shown to offer significant potential in the biological and technological fields. BNPs exist in different forms, which include alloys, core-shell and aggregates. The popularity of this group of NPs stems from their superior optical, catalytic, electronic, and thermal properties, among others [1]. At the same time, the combination of noble metals such as gold and platinum has been favored in nanomedicine, mainly as anticancer drug delivery vehicles [2,3,4]; BNPs such as silver-selenium nanocomposites have recently emerged as excellent alternatives in cancer theranostics [5].

Over the last few decades, silver (Ag) and selenium (Se) NPs have been synthesized by several techniques using top-down and bottom-up approaches. These NPs have clearly shown desirable activity, including antioxidant, anticancer, catalytic, and antibacterial activity. Selenium is an essential micronutrient in biological systems and a structural element of selenocysteine. This amino acid is a component of glutathione peroxidase; hence, Se is widely regarded as an antioxidant. SeNPs possess the favorable properties of the Se and are regarded as the better Se species due to their excellent biological, anticancer, and therapeutic properties [6]. AgNPs are especially noted for their excellent conductivity and antimicrobial activity that has been exploited in medicine for use in surgical prostheses wound healing, and dental implants [7].

Moreover, the general ease of synthesis and a good surface-area-to-volume ratio of these NPs has further prompted their application as nanocarriers. However, both Ag and Se are also reported to pose some challenges with regard to cytotoxicity and genotoxicity in normal cells [8,9,10], especially at high concentrations. The combination of Ag and Se NPs results in the continuous tuning of the AgNPs and SeNPs in the ultraviolet, visible and near-infrared region to obtain BNPs with improved functional properties compared to the individual mono-metallic nanoparticles [11,12,13]. These hybrid NPs can be synthesized using chemical reductants (citrate, sodium borohydride, hydrazine, dimethylamine borane, and hydroquinone) or green synthesis routes. Synthesis via chemical reduction has been the most commonly reported approach, with a limited number of studies reporting on the synthesis of Ag-Se NPs using green chemistry [14,15,16,17].

The extensive use of chemical techniques might be attributed to the fact that they generate a high yield of NPs and allows for easy process optimization and strict control of the NPs’ dimensions and morphology. Despite this advantage, there are serious concerns about using chemical reductants in NP synthesis due to their toxicity, high cost, and detrimental environmental effect. The current study adopted a biogenic approach to synthesize core-shell Ag@Se BNPs using *Ocimum tenuiflorum* extract due to its comparative advantage of ecological safety and mild reaction conditions. Aqueous extracts of *Ocimum tenuiflorum* have demonstrated significant capacity to reduce inorganic ions and concurrently act as capping agents in NPs. Hence, they have been employed here to synthesize the Ag@Se BNPs [18,19,20]. The extract contains beneficial compounds with significant biological activities, which may confer additional bioactivity on the synthesized NPs [21].

The two major challenges faced in the green synthesis of NPs using plant extracts are that the NPs’ size and shape may not be easily reproduced and that the synthesis technique cannot be applied with ease on a large scale [22]. Different studies have shown that several factors such as type of plant extract, extract volume, reactant concentration, pH, and temperature, can affect the synthesis process. Most studies on controlled NP synthesis have considered each of these components singularly to understand how they affect the structure of the NPs [23]. Furthermore, to mitigate cytotoxicity and improve biocompatibility, most Ag or SeNPs are functionalized with biodegradable polymers. It has been reported that citrate-capped AgNPs produced a time- and dose-dependent cytotoxicity. Upon functionalization of the NP surface with oligo (ethylene glycol) (OEG)-alkanethiol, the cellular uptake and cytotoxicity were reduced in mouse embryonic fibroblasts. This study highlighted the importance of a preformed protein corona on the AgNPs surface, especially for cellular uptake and cytotoxicity [24]. It was further noted that the tuning of the surface functionalities of NPs controlled their ability to adsorb serum proteins and thereby regulated their biological properties. This is important to take into consideration when designing nano-delivery systems [25]. However, rather than a single factor, multiple factors contribute to the design of the NPs. In this study, by keeping other variables constant, the effect of the extract volume and reaction temperature on the structure and properties of these NPs was considered. The design of experiment-based optimization using response surface methodology was used to map the response surface region and determine the extract volume and temperature condition required for optimal NP synthesis.

Synthesis of NPs through biogenic approaches has been reported to confer properties such as antioxidant, anticancer, and biocompatibility features on the NPs [26,27,28,29]. A critical understanding of the properties of green-synthesized NPs is essential to decipher its potential application fully. In the current study, the antioxidant, cytotoxic, and genotoxic assessment of the NPs was further carried out.

## 2. Materials and Methods

### 2.1. Materials

Silver nitrate (AgNO_3_), ascorbic acid, sodium selenite (Na_2_SeO_3_), trichloroacetic acid (TCA), sodium carbonate, 3-(4,5-dimethyl thiazol-2-yl)-2,5-diphenyl tetrazolium bromide (MTT), phosphate-buffered saline (PBS), and gallic acid were supplied by Merck (Darmstadt, Germany). Folin-Ciocalteau reagent, 2,2-diphenyl-1-picrylhydrazyl (DPPH), and potassium ferricyanide, iron (III) chloride were obtained from Sigma Aldrich Chemical Co. (St Louis, MO, USA). Eagle’s Minimum Essential Medium (EMEM), fetal bovine serum (FBS), trypsin-versene, and antibiotics (Penicillin (5000 units/mL)/Streptomycin (5000 µg/mL)) were purchased from Lonza Bio-Whittaker (Verviers, Belgium). *Ocimum tenuiflorum* was collected within the province of KwaZulu-Natal, South Africa, and identified at the Department of Botany, University of KwaZulu-Natal. A voucher specimen (K. Olofinsan and F. Olawale 2) was deposited in the Ward Herbarium of the university. The breast adenocarcinoma (MCF-7) and embryonic kidney (HEK293) cells were obtained from the ATCC, Manassas, VA, USA. Sterile plasticware for cell culture was purchased from Corning Inc. (New York, NY, USA). Ultrapure (18 MOhm) water (Millipore, France) was used throughout.

### 2.2. Plant Extraction and Gas Chromatography-Mass Spectrometry (GC-MS) Analysis

The inflorescence of the fresh sample of *Ocimum tenuiflorum* was collected, air-dried at room temperature, and mechanically pulverized. The aqueous extract of the plant was obtained by dissolving 5 g of the sample into 50 mL of 18 MΩ Milli-Q water and incubating it at 70 °C for 2 h. The mixture was allowed to cool before successive filtration (3×) using an ultra-fine filter paper (0.7 µm pore size). Part of the extract obtained was used for the subsequent phase of NP synthesis, while the remaining portion was lyophilized and stored at room temperature for characterization.

The dried aqueous extract was subjected to gas chromatography-mass spectrometry (GC-MS) analysis, using a single quadrupole Shimadzu GCMS-QP2010SE gas chromatograph-mass spectrometer connected to the National Institute of Standards and Technology (NIST) library (Gaithersburg, MD, USA). The instrument enables direct sample injection and easy expandability without any changes to the gas chromatograph. An SH-Rxi-5Sil MS capillary column (30 m, 0.25 mm, 0.25 μm; Shimadzu, Shiga, Japan) was used as the stationary phase. The setup parameters were the following: column oven temperature of 60 °C, injection temperature of 250 °C, column pressure of 59.7 kPa for the helium gas carrier, column flow of 1.03 mL/min, the linear velocity of 37.0 cm/s and injection volume of 1 µL made in split mode with a split ratio of 20:1. The mass spectrometer was run in full scan using the electron ionization mode. Base peaks were obtained at a scan speed of 2500 µ/s within the 50–700 atomic mass unit scan range. The peaks were interpreted by comparing the molecular fragmentation pattern and retention time with compounds with similar spectral data on the NIST database.

### 2.3. Biogenic Synthesis of Ag@Se NPs

The synthesis of Ag-core Se-shell NPs was adapted from the protocol described by Sibiya and Moloto with significant modifications [16]. Briefly, 5 mL of the crude aqueous extract of *Ocimum tenuiflorum* was added to 50 mL of 1 mM AgNO_3_ in a conical flask. The reaction mixture was continuously stirred for about 2 h at 50 °C to initiate the synthesis of the Ag core of the NPs. The Se precursor (50 mL of 1 mM Na_2_SeO_3_) was then added, followed by the addition of 5 mL of the aqueous extract. The reaction mixture was stirred continuously for 4 h at 80 °C, then centrifuged at 6000 rpm for 15 min, and the supernatant was discarded. The pellet was reconstituted in 18 MΩ Milli-Q water.

### 2.4. Generation of a NP Synthesis Model and Optimization of Physicochemical Parameters

Response surface methodology of the Ag@Se NPs was performed using a central composite design to determine the effect of two primary independent variables (volume of extract and reaction temperature) on the zeta potential, hydrodynamic size, and NP concentration. Using the Minitab statistical software package (Minitab v.16, Minitab Inc., State College, PA, USA), thirteen experimental tests with five repeated center points (*X_1_* = 1000 µL and *X_2_* = 750 °C) were designed. The low and high limit of the extract used was set at 500 µL and 1500 µL, while 50 °C and 100 °C were selected as the minimum and maximum temperature, respectively. A second-order polynomial Equation (1) correlated the independent NPs’ synthesis parameter variables to the dependent variables.

(1) $$Y=\alpha_{0}+\alpha_{1}X_{1}+\alpha_{2}X_{2}+\alpha_{11}X_{1}^{2}+\alpha_{22}X_{2}^{2}+\alpha_{12}X_{1}X_{2}$$

where *X_1_* = volume of extract in µL, *X_2_* = reaction temperature, *Y* = dependent/response variables, α_0_ = constant, α_1_ and α_2_ = linear co-efficient, α_11_ and α_22_ = quadratic co-efficient, and α_12_= interaction/cross product co-efficient. The significant impact of the independent variables on the *Y* variables was analyzed statistically using analysis of variance based on *p*-value (95% confidence interval) and F ratio. A response surface curve was generated for each dependent variable and used to predict the optimal condition required for the efficient synthesis of the NPs using a numerical desirability function. The generated model was evaluated by comparing how well the observed data fit into the quadratic model generated using statistical parameters such as correlation coefficient (r^2^) and adjusted correlation coefficient (r^2^ adj).

### 2.5. Nanoparticle Characterization

The surface plasmon resonance of the NPs was verified using UV-visible spectroscopy (Jasco V-730 UV-visible/ NIR Bio spectrophotometer, JASCO Corporation, Hachioji, Japan). NP solutions were diluted before analysis, and spectra were obtained at room temperature. High-resolution transmission electron microscopy (HR-TEM) analysis of the NPs was carried out using a camera attached to a JOEL TEM 1400 instrument (Jeol, Tokyo, Japan) operating at 100 kV. NP sizing was performed using the inbuilt Image J software version 1.8.0. (on a sample field of fifty NPs. For scanning electron microscopy (SEM), the NPs were affixed using double-sided carbon tape (Nisshin EM, Co Ltd., Tokyo, Japan) onto a SEM stud and coated with gold particles using a Polaron SC500 Sputter Coater (Quorum Technologies, Ashford, UK). The stud was placed in a LEO 1450 SEM and analyzed using SmartSEM software version 5.03.05 (ZEISS, Jena, Germany).

Functional groups present on the surface of the NPs were determined using Fourier transform infrared (FTIR) spectroscopy (PerkinElmer Inc. Waltham, MA, USA). In contrast, energy dispersive x-ray (EDX) spectroscopy (Oxford Instruments, Oxfordshire, UK) was used to analyze the elemental constituents of the NPs.

The mean hydrodynamic size, concentration, polydispersity and zeta potential of the NPs were measured using the nanoparticles tracking analysis (NTA, Nanosight NS500, Malvern Instruments, Worcestershire, UK). The NPs synthesized were diluted 1:1000 with 18 ΩM water, and 1mL of the diluent was analyzed. The total NP concentration (TNC) per ml was obtained using the equation below:

TNC = NPs per mL × dilution factor(2)

The NPs’ polydispersity index (PDI) is a dimensionless quantity that varies with the mean size and standard deviation of the NPs. For the Gaussian distribution assumption, PDI is derived from Equation (3) [30].

(3) $$\mathrm{PDI}={\left(\frac{\sigma }{d}\right)}^{2}$$

where *σ* = standard deviation (SD) and *d* = mean NP diameter.

### 2.6. Antioxidant Activity

The NPs’ antioxidant properties were assessed using the 2,2-diphenyl-1-picrylhydrazyl (DPPH) radical and ferric ion scavenging assays. All experiments were conducted in triplicate. The DPPH assay was performed using a protocol adapted from Bukhari et al. [31]. Different concentrations of the test and standard ascorbic acid (20–100 µg/mL) sample was added to 0.3 mM DPPH solution in a 2:1 (*v*/*v*) ratio. The mixtures were incubated in the dark at room temperature, and the absorbance was measured at 517 nm against a water blank. The DPPH radical scavenging activity was expressed as a percentage and calculated using Equation (4).

(4) $$\%\;\mathrm{DPPH}\;\mathrm{scavenging}\;\mathrm{activity}=100\;\times \;(1-\frac{\mathrm{sample}\;\mathrm{absorbance}}{\mathrm{absorbance}\;\mathrm{of}\;\mathrm{control}})$$

Ferric ion reduction was also used to assess the NPs’ antioxidant potential using a protocol described by Oyaizu et al. with minor modifications [32]. An equal volume (50 µL) of each sample (NPs, plant extract, and ascorbic acid standard) were added to 50 µL phosphate-buffered saline and 50 µL of 1% potassium ferricyanide. The mixture was incubated at 50 °C for 30 min and then acidified with 50 µL trichloroacetic acid (10%). Thereafter, the mixture was centrifuged at 3000 rpm for 10 min, and 50 µL of the supernatant was added to 50 µL 18 MΩ Milli-Q water and 25 µL FeCl_3_ (0.1%). The absorbance of the mixture was read at 700 nm, and ferric ion reducing power was expressed using Equation (5).

(5) $$\mathrm{Ferric}\;\mathrm{ion}\;\mathrm{reducing}\;\mathrm{power}=\frac{\mathrm{absorbance}\;\mathrm{of}\;\mathrm{sample}}{\mathrm{absorbance}\;\mathrm{of}\;\mathrm{gallic}\;\mathrm{acid}}\;\times \;100\%$$

### 2.7. Cytotoxicity

The cytotoxic effect of the Ag@Se NPs on the human embryonic kidney (HEK293) and breast adenocarcinoma (MCF-7) cells was determined using the 3-(4,5-dimethyl thiazol-2-yl)-2,5-diphenyl tetrazolium bromide (MTT) colorimetric assay. All cells were cultured in minimum essential medium (EMEM, 200 µL) containing 10% fetal bovine serum (FBS) and 1% antibiotics, and incubated at 37 °C under a 5% CO_2_-saturated humidified atmosphere. The cells were seeded at a cell density of 1.8–2.2 × 10^4^ cells/well in 48-well plates and incubated for 24 h to allow the cells to attach. The medium was subsequently replenished, followed by adding the NPs and the standard drug 5-fluorouracil (5-FU). After incubation for 48 h, the medium was removed, and MTT reagent (5 mg/mL in PBS) together with the complete medium were successively added into each well in a 1:10 (*v*/*v*) ratio. Cells were then incubated for 4 h at 37 °C. Thereafter the medium was removed, and cells were washed with PBS, followed by the addition of 100 µL dimethyl sulfoxide (DMSO). The plates were then gently shaken for 5 min at 55 rpm to dissolve the formazan crystals produced by metabolically active cells. The absorbance was read at 570 nm, and the cytotoxic effect was expressed in terms of cell viability as in Equation (6).

(6) $$\%\;\mathrm{cell}\;\mathrm{viability}\;=\;\frac{\mathrm{optical}\;\mathrm{density}\;\mathrm{of}\;\mathrm{test}\;\mathrm{sample}}{\mathrm{optical}\;\mathrm{density}\;\mathrm{of}\;\mathrm{control}}\times 100$$

### 2.8. Apoptosis

The apoptosis assay was used to support the MTT assay results and evaluate the possible mechanisms of cell death. The acridine orange/ethidium bromide (AO/EB) (100 µg/mL equimolar volume in PBS) dual staining method was used to determine apoptotic cell death in vitro [33]. Cells were plated as in 2.7 and incubated for 24 h, after which the medium was removed. Subsequently, 200 µL fresh medium was added to each well, followed by 100 µg/mL of the NPs and 5-FU, and incubated for 24 h under 37 °C. The medium was then removed, and cells were washed with 200 µL PBS before staining with 10 µL of AO/EB dye. Plates were rocked at 55 rev/min on a platform rocker for 5 min, then rinsed with PBS and examined under a fluorescence microscope and images captured with a CC12 fluorescence camera (Olympus Co., Tokyo, Japan) at 200× magnification.

### 2.9. Allium Cepa Toxicity

NP toxicity was further assessed using an adaptation of the *Allium cepa* bioassay previously described [34,35,36]. Briefly, fifty onion bulbs (45 ± 5 g) were rooted in distilled water and allowed to grow for 48 h in a growth room under 24 h in the dark. Onion bulbs with root lengths of 2.0–3.0 cm were treated with different concentrations (1, 10 and 100 µg/mL) of the NPs. Treatment with distilled water and 300 mM hydrogen peroxide (H_2_O_2_) were used as negative and positive controls, respectively. The experiments were conducted in triplicates, and the water and NP treatment suspensions were changed every 24 h to prevent fungal growth. The root lengths of the onion were measured daily for 48 h for root growth inhibition. After 48 h of treatment, the roots of the negative control and NP treated groups were harvested and fixed in ethanol: glacial acetic acid (3:1 *v*/*v*) and 2.5% glutaraldehyde (in 0.1 M phosphate buffer) for microscopic analysis of cytogenotoxicity.

#### 2.9.1. Cytogenotoxocity Studies

Roots were removed from the ethanol/glacial acetic fixative, and about 1–2 mm of the tip was cut off with a sharp scalpel on a glass slide. The excess fixative was blotted, and 1 N HCl was added to each root tip for about 5 min. The excess HCl was then removed, and a single drop of 2% toluidine blue dye was added. After 5 min the root tip was gently macerated with a coverslip and sealed with nail varnish for microscopic examination (1000× magnification). A total of 1000 cells in each onion bulb were examined for cell division and chromosomal aberration (including vagrant chromosome, c-metaphase, binuclei, sticky chromosome and chromosomal lags) and the data were expressed as a percentage. Images of the *Allium cepa* root cells were obtained using an Olympus Microscope (Olympus Co., Tokyo, Japan). The mitotic index, mitotic depression and chromosomal aberration index were determined using Equations (7)–(9).

(7) $$\mathrm{Mitotic}\;\mathrm{index}=\frac{\mathrm{Number}\;\mathrm{of}\;\mathrm{dividing}\;\mathrm{cells}}{\mathrm{Total}\;\mathrm{number}\;\mathrm{of}\;\mathrm{cells}}\times 100$$

(8) $$\mathrm{Mitotic}\;\mathrm{depression}=\frac{\mathrm{Mitotic}\;\mathrm{index}\;\mathrm{of}\;\mathrm{control}-\mathrm{Mitotic}\;\mathrm{index}\;\mathrm{of}\;\mathrm{test}}{\mathrm{Mitotic}\;\mathrm{index}\;\mathrm{of}\;\mathrm{control}}\times 100$$

(9) $$\mathrm{Chromosomal}\;\mathrm{aberration}\;\mathrm{index}=\frac{\mathrm{Total}\;\mathrm{number}\;\mathrm{of}\;\mathrm{altered}\;\mathrm{cells}}{\mathrm{Total}\;\mathrm{number}\;\mathrm{of}\;\mathrm{counted}\;\mathrm{cells}}\times \;100$$

#### 2.9.2. Electron Microscopy Studies

The normal control and the 100 µg/mL Ag@Se NP treated *Allium cepa* root were used in electron microscopy studies. The samples were removed from buffered 2.5% glutaraldehyde and washed 3× in phosphate buffer. The tissues were then post-fixed in 0.5% osmium tetraoxide for 1 h and washed 3x in phosphate buffer to improve staining contrast and to ensure stability during dehydration, embedding, and electron bombardment [37]. The tissue was then subjected to successive dehydration in 30%, 50%, 75%, and 100% acetone followed by exposure to resin and acetone for 18–24 h and embedded in a mold filled with whole resin for 8 h at 70 °C for polymerization. Finally, ultra-thin sections were obtained for electron microscopy examination. TEM was undertaken and images captured as described in Section 2.5.

### 2.10. Molecular Modelling with DNA

The compounds identified from the GCMS analysis were used for molecular docking with DNA. The 2D structures of the ligands were obtained from the PubChem database (https://pubchem.ncbi.nlm.nih.gov, accessed on 15 June 2021), and right-handed double helix DNA (B-DNA) structure was obtained from protein data bank (PDB ID: 1bna) (https://www.rcsb.org/, accessed on 15 June 2021). Molecular docking analysis was performed using the maestro tool on the Schrodinger suite [38]. The 2D structure of the compounds identified from the extract was first downloaded from the PubChem database and converted to low-energy 3D structures suitable for ligand docking. Ligand preparation was achieved using the LigPrep panel of the maestro tool available on the Schrodinger suite. The compounds were optimized in the OPLS3 force field. The ionization state was generated at pH 7 using Epik. The desalt ligand option was selected to remove extra structures such as water molecules and counter ions that may be present from the database. In addition, the retain specified chirality and generate low-energy ring conformation options were selected.

Since structures available on the PDB database may contain sub-structures such as water molecules, metal ions, and co-factors, which may alter the docking result, the DNA structure downloaded was initially prepared using the protein preparation wizard panel of the maestro tool. The protein preparation step involved an initial pre-processing stage in which hydrogen bonds were added, missing residues and loops were filled using the prime option, water molecules were deleted, and the appropriate bond orders were assigned. Subsequently, the hydrogen bonding network was optimized using the PROPKA panel, which re-orientates the hydrogen atoms and water molecules. Finally, the water present in the binding sites was removed, and the entire structure was minimized in the OPLS3 force field.

Following protein preparation, the binding site on the DNA molecule was predicted using a site map panel to detect all the possible binding sites on the structure. The quantitative measure of the druggable site for docking was given by site score. The site with the highest score (map score of 0.99) was used to generate a receptor grid for standard precision and extra-precision docking. The standard precision and extra-precision docking are forms of glide (grid-based ligand docking and energetics) docking that uses a different scoring function to calculate the affinity of the compounds for the DNA structure. The extra-precision docking is more accurate than the standard precision docking because it carries out more conformational sampling within the DNA structure’s binding pocket and compensates for unfavorable interaction that might result in false positives. The 2- dimensional molecular interaction obtained from the XP precision docking was used to assess the possible forms of bonding and non-bonding interactions between the compounds and the DNA. The result of the molecular docking studies was compared with quinacrine, a DNA-binding anticancer agent [38,39,40].

### 2.11. Statistical Analysis of Data

All quantitative data were analyzed using graph pad prism statistical software version 6.01 (GraphPad Software, La Jolla, CA, USA). Comparison between different treatment groups and control were conducted using one-way analysis of variance at 95% confidence interval.

## 3. Results

### 3.1. Nanoparticle Synthesis and Characterization

**Figure 1:**
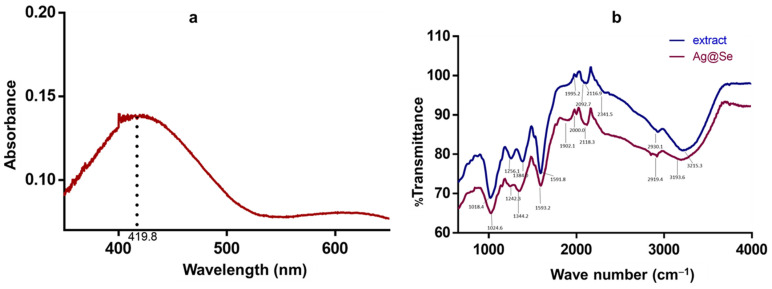
(**a**) Ultraviolet-visible spectrum of the *Ocimum tenuiflorum* synthesized Ag@Se nanoparticles, and (**b**) FTIR spectra of the *Ocimum tenuiflorum* extract and green-synthesized Ag@Se nanoparticles.

Figure 1 depicts the UV-vis and FTIR spectra of the synthesized NPs. The formation of Ag@Se NPs was initially confirmed by absorbance maxima observed around 420 nm (Figure 1a). The FTIR spectra confirmed the presence of the functional groups present in the NPs, which coincided with the peaks of the plant extract (Figure 1b). Thus, peaks observed at 1024.6, 1344.2, 1593.2, 1902.1, 2118.3 and 3193.6 can be attributed to the C=O stretch of the ketone carbonyl, O–H stretch of carboxylic acid, N–H bend of primary amines, C=O stretch of carbonyl on transition metal complexes, C=N stretch of nitriles and the O–H stretch of phenol, respectively [41,42].

**Figure 2:**
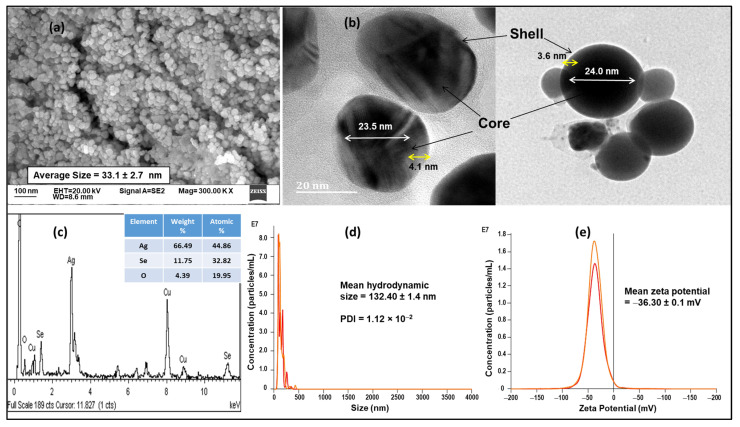
(**a**) Scanning electron microscopy (SEM) image of Ag@Se NPs, (**b**) High resolution-transmission electron microscopy (HR-TEM) image of Ag@Se NP core-shell, (**c**) HR-TEM- energy dispersive x-ray (EDX) spectrum of Ag@Se NPs by weight and atomic percent, (**d**) Ag@Se NP concentration and size from nanoparticle tracking analysis (NTA) and, (**e**) Ag@Se NP concentration and zeta potential from NTA.

Further characterization of the plant via GC-MS analysis revealed eight tentative compounds, including dimethyl silane diol, catechol, diethyl phthalate, caffeine, l-(+)-ascorbic acid 2,6-dihexadecanoate, stigmasterol, cholesta-4,6-dien-3beta-ol and stigmastan-3,5-diene (Table 1, Supplementary Figures S1 and S2). The NPs formed had near-spherical, pentagonal, and hexagonal shapes with a dense core of Ag (23.5–24 nm) and a Se shell (3.6–4.1 nm), as evidenced from HR-TEM. The Ag@Se NP size was in the order of 33.1 ± 2.7 nm as observed under SEM (Figure 2). In addition, NTA results (Figure 2d,e) revealed that the NPs were uniformly dispersed in the aqueous suspension with a PDI of 1.12 × 10^−2^ and an average size of 132.4 ± 1.4 nm. The NPs elemental analysis via EDX showed the presence of Ag and Se as the main constituents with an atomic percentage composition of 44.86% and 32.82%, respectively (Figure 2). The Ag peak was observed around 3.2 KeV, and Se peaks around 1.4 and 11.2 KeV.

### 3.2. Model-Based Optimization of Physicochemical Conditions for NP Synthesis

Thirteen experimental runs were carried out based on the central composite design matrix described earlier, and the results are provided in Table 2.

Based on the conducted experiments, the following quadratic models were developed to explain the relationships between the independent variables (amount of extract and reaction temperature) and dependent variables (zeta potential, hydrodynamic size, and NP concentration).

(10) $$\mathrm{Zeta}\;\mathrm{potential}=-65.0+0.0400\;x_{1}+0.643\;x_{2}-0.000006\;x_{1}^{2}-0.00211\;x_{2}^{2}\;-0.000384\;x_{1}x_{2}$$

(11) $$\mathrm{Hydrodynamic}\;\mathrm{size}=86.5+0.0092\;x_{1\;}-0.538\;x_{2}+\;0.000004\;x_{1}^{2}+0.00156\;x_{2}^{2}\;-0.000122\;x_{1}x_{2}$$

(12) $$\mathrm{Concentration}=-4.01+0.00328\;x_{1}+0.0222\;x_{2}-0.000003\;x_{1}^{2}\;+0.000300\;x_{2}^{2}+0.000056\;x_{1}x_{2}$$

$x_{1}$ and $x_{2\;}$ are independent variables. $x_{1}$ represents the volume of extract, while $x_{2\;}$ represents the temperature. By varying the amount of extract and temperature the zeta potential, hydrodynamic size and nanoparticle concentration can be controlled based on the equation above.

The regression coefficient (r^2^ = 0.75, 0.68 and 0.96) reflected a positive correlation between the independent variables (amount of extract and reaction temperature) and dependent variables (zeta potential, hydrodynamic size, and nanoparticles’ concentration) (Supplementary Table S1). However, the adjusted r^2^ value gave a comparatively lower correlation coefficient with values of 0.56, 0.44, and 0.93 for the zeta potential, hydrodynamic size and NP concentration, respectively. Statistical analysis of the model produced a *p*-value < 0.05 for the zeta potential and concentration as response variables. The F-value of the three models generated also supported the model’s validity with large (>1.0) F ratios of 4.16, 2.91 and 35.14, which implied that the variation was less likely to be due to a chance correlation.

**Figure 3:**
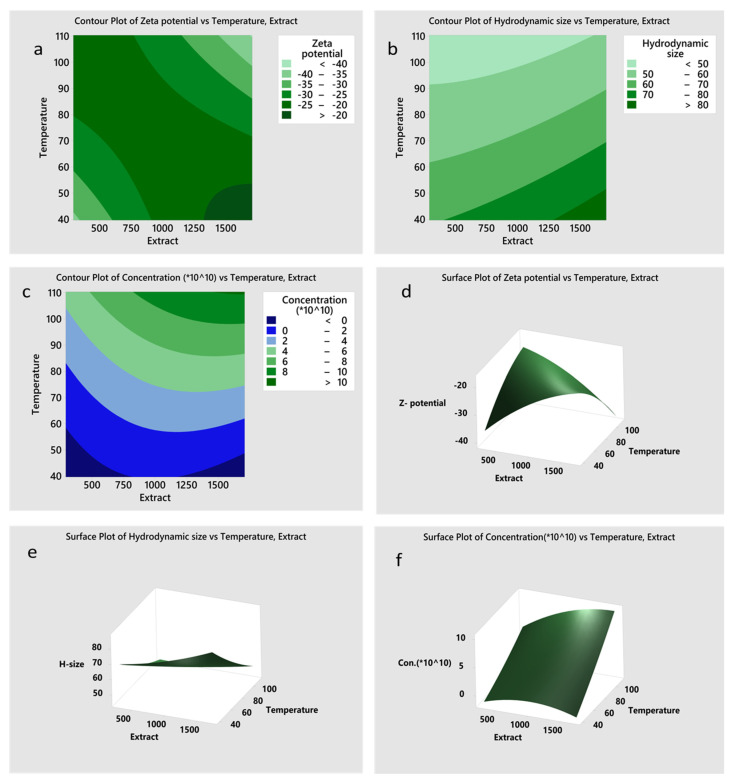
Contour plots (**a**–**c**) and 3D surface response plots (**d**–**f**) depicting the effects of volume of extract and temperature on zeta potential (**a**,**d**), hydrodynamic size (**b**,**e**), and nanoparticles synthesis (**c**,**f**).

Response surface mapping for the dependent variables is illustrated in Figure 3 using 3D surface response plots and 2D contour plots. The zeta potential and NP concentration surface response plots suggested a curvilinear relationship with the independent variables. In contrast, the response plots for the hydrodynamic size was roughly linear, implying that one of the independent variables (temperature) was the overriding determinant of the particle size. As illustrated in Table 3, optimal NP synthesis with favorable stability was achieved at a temperature of about 110.36 °C and extract volume within the range of 0.5–1.7 mL.

### 3.3. Antioxidant Activity and Cytotoxicity

**Figure 4:**
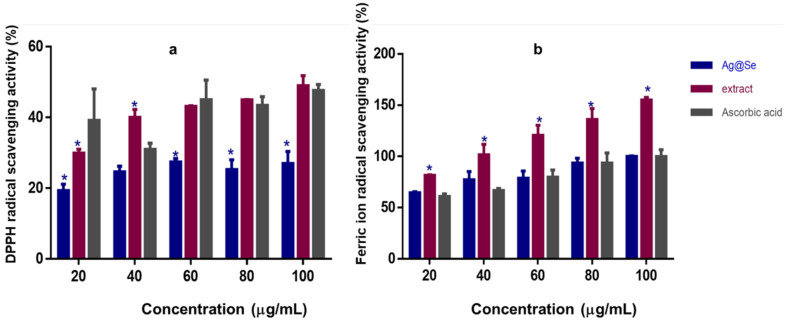
(**a**) 2, 2-diphenyl-1- picrylhydrazyl (DPPH) radical scavenging activity, and (**b**) ferric ion scavenging activity of *Ocimum tenuiflorum* extract and Ag@Se NPs compared to ascorbic acid. Data are presented as mean ± SD (*n* = 3). * *p* < 0.05 for statistical significance compared to standard antioxidant (ascorbic acid).

As seen in Figure 4, Ag@Se NPs showed a dose-dependent DPPH and ferric ion scavenging activity. It was noted that the NPs had a higher capacity to scavenge ferric ions than DPPH radicals (Table 4). However, the extract showed more radical scavenging activity than the NPs and ascorbic acid standard (Figure 4, Table 4).

**Figure 5:**
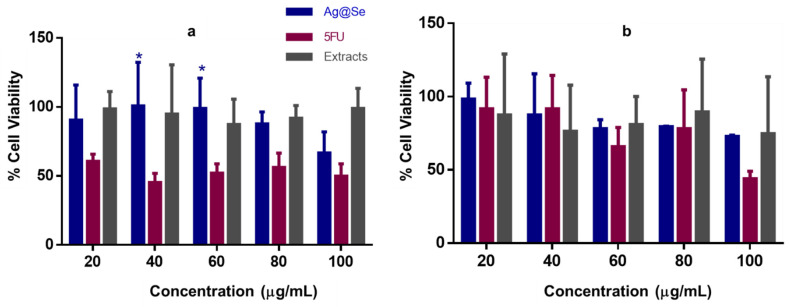
Cytotoxicity assay of Ag@Se NPs,5-FU and the plant extract in (**a**) HEK293 and (**b**) MCF-7 cells. Data are represented as mean percentage cell viability ± SD (*n* = 3). * *p* < 0.05 when compared to the 5-FU standard.

The result in Figure 5 suggests that in addition to the antioxidant activity, at high concentrations exceeding 60 µg/mL, Ag@Se NPs demonstrated significant dose-dependent cytotoxicity in the HEK293 and MCF-7 cells. Interestingly, the NPs were less cytotoxic to the HEK293 cells than the MCF-7 cells (Table 5), which could be due to the biocompatibility of the plant extract. The plant extract itself showed little or no cytotoxicity in both cell lines justifying its use in the NP synthesis.

### 3.4. Apoptosis

**Figure 6:**
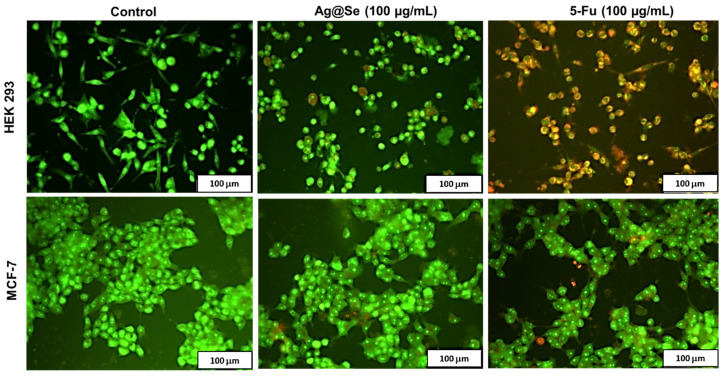
Fluorescent images of acridine orange/ ethidium bromide dual stained human embryonic kidney (HEK 293) and breast adenocarcinoma (MCF-7) cells. Green fluorescence denotes viable cells, dark red denotes necrotic cells, yellow to orange denotes cells undergoing late apoptosis, while yellow-green nuclei denote early apoptotic cells. Scale bar = 100 µm.

Fluorescence microscopy using acridine orange/ethidium bromide dual staining was adopted to study cell death induced by the Ag@Se NPs. The untreated HEK293 and MCF-7 cells exhibited green fluorescence with normal nuclear and cytoplasmic morphology typical of viable cells (Figure 6). A similar observation was observed in the Ag@Se NP treated cells, with only a few cells in the early and late apoptosis stage. On the other hand, 5-FU-treated HEK293 cells showed a significant population of cells with fragmented nuclei (red fluorescence) and yellowish-green fluorescent cells with membrane blebs, which are characteristic features of necrotic and late apoptotic cells. Hence, together with the cytotoxicity studies, the results suggest that Ag@Se NPs are biocompatible at low concentrations, but induce apoptosis in some normal and cancer cell lines at 100 µg/mL.

### 3.5. Allium cepa Assay

**Figure 7:**
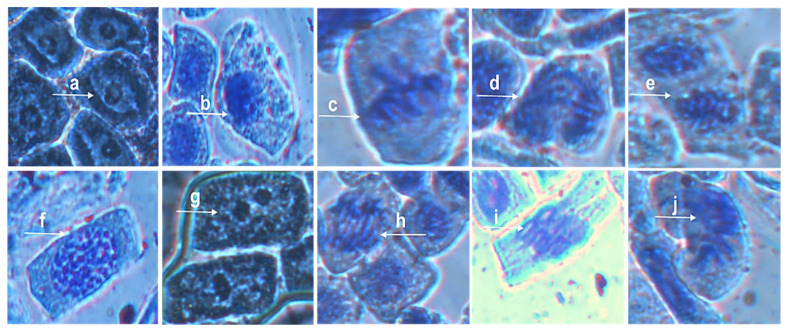
Genotoxicity studies on *Allium cepa* root cells. Control cells in interphase, prophase, metaphase, anaphase, and telophase are labeled (**a**–**e)**, respectively. (**f**) = vagrant chromatin fragment in nucleus, (**g**) = binuclei, (**h**) =Anaphase bridge with lag, (**i**) = sticky chromosome, (**j**) = c-metaphase.

**Figure 8:**
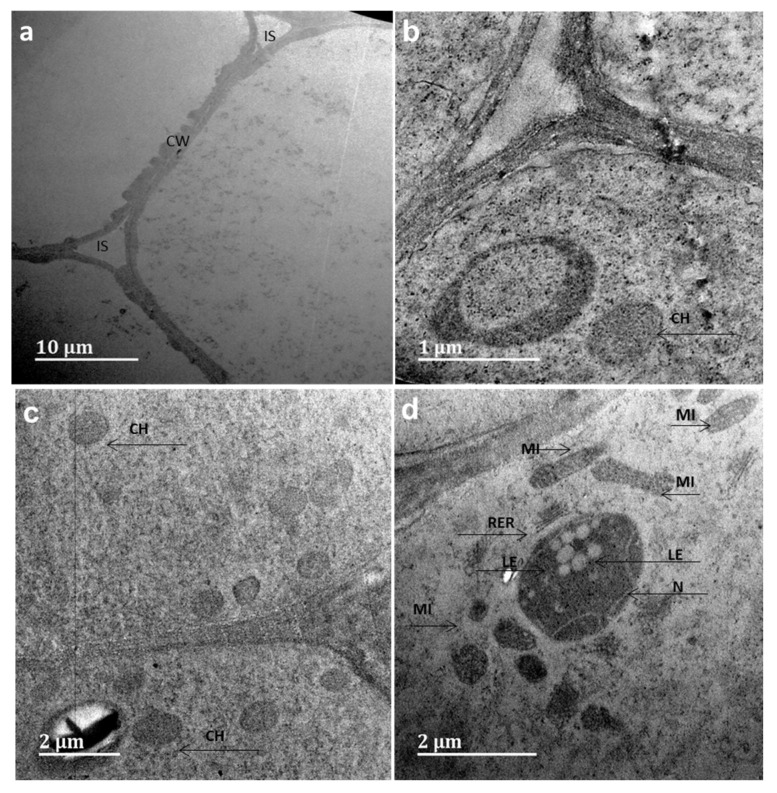
TEM of the ultrastructure of meristematic cells of the control (**a**,**b**) and Ag@Se (**c**,**d**) treated *Allium cepa* root. S = intracellular space, CW = cell wall, CH= chloroplast, MI= mitochondria, N = nucleus, RER = rough endoplasmic reticulum, LE = lesions.

The *Allium cepa* bioassay was used to further verify the biocompatibility of the NPs at different concentrations. Exposure of *Allium cepa* root to Ag@Se NPs at concentrations of 1 µg/mL and 10 µg/mL over 48 h did not show any significant alteration in the growth rate (Supplementary Figure S3). However, a slight decrease in growth rate was observed at a concentration of 100 µg/mL. Furthermore, the addition of 300 mM H_2_O_2_ showed more significant growth inhibition compared to the NPs. Following 48 h of exposure to the Ag@Se NPs, the rate of mitotic cell division in the *Allium cepa* root significantly decreased in a dose-dependent manner (Table 5). The *Allium cepa* root cells also showed significant cytogenetic aberrations with increasing NP concentration (Table 6). Major genetic aberrations observed include vagrant chromatin fragments in the nucleus, binuclei, anaphase bridge with lags, sticky chromosome, and c-metaphase (Figure 7). Moreover, TEM evaluation of the root revealed notable changes such as an abundance of cellular structure and nuclear lesions in the Ag@Se NP treated *Allium cepa* cells compared to the control (Figure 8).

### 3.6. Molecular Docking Studies

**Figure 9:**
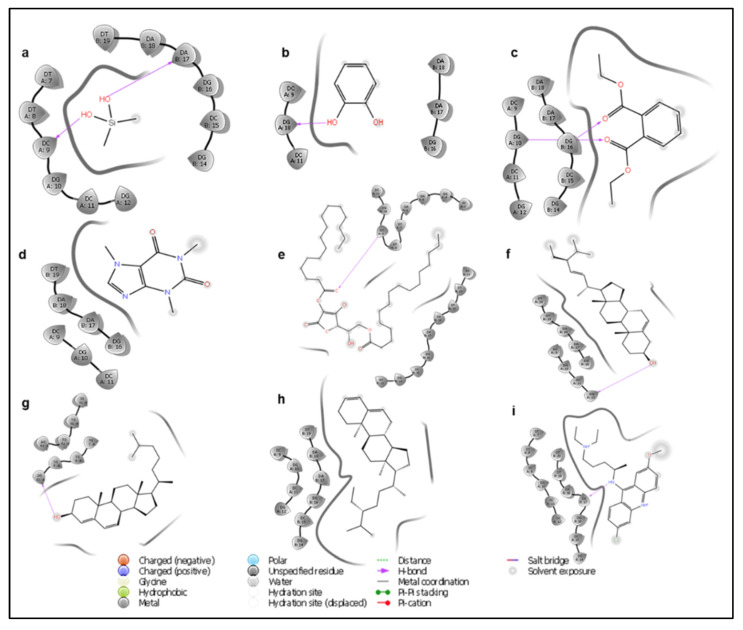
2D ligand interaction diagram of dimethyl silanediol (**a**), catechol (**b**), diethyl phthalate (**c**), caffeine (**d**), l-(+)-ascorbic acid 2,6-dihexadecanoate (**e**), stigmasterol (**f**), cholesta-4,6-dien-3beta-ol (**g**), stigmastan-3,5-diene (**h**), and quinacrine (**i**) with B-DNA.

Molecular docking analysis was used to determine the level of interaction between the compounds present in the plant extract and DNA as a measure of possible genotoxic activity. From the standard precision docking score, caffeine had the best binding free energy of interaction, forming hydrogen bonds with the deoxycytidine base on the A-strand of DNA (Table 7, Figure 9 and Supplementary Figure S4). However, the extra precision docking showed that dimethylsilaniol had the best binding energy of interaction with a docking score value of −4.22 kcal/mol. Conversely, compounds such as cholesta-4,6-dien-3beta-ol and stigmastan-3,5-diene showed a positive binding free energy of interaction from the XP docking, which suggested that the compounds could not spontaneously interact with the DNA molecule. Compared to the standard DNA intercalating agent (quinacrine) used, the compounds found in the plant extract had a much weaker free energy of interaction, suggesting that they posed a lower genotoxic threat. In addition, most of the compounds had very weak non-bonding interactions with the nucleic acid bases on the DNA molecule.

## 4. Discussion

BNPs consisting of Ag and Se are among some of the promising NPs that have been synthesized. As chalcogenides, they show interesting optical and electronic properties, which have attracted significant research interest. Previous studies have reported the successful synthesis of silver selenide (Ag_2_Se) using chemical techniques that required harsh reaction conditions [17,43,44]. The current study demonstrated the successful synthesis of Ag@Se NPs using *Ocimum tenuiflorum* extract. AgNO_3_ was initially reduced by the extract to form the core of the NPs, while the shell (Se) was formed by the reduction of Na_2_SeO_3_. The NP formation was confirmed by an absorbance maximum close to 420 nm. Similar observations have also been reported during the formation of Ag_2_Se NPs using tri-n-octyl-phosphine as reductant and hexadecylamine as capping agent [45]. In the present study, rather than using different reductant and capping agents, the extract demonstrated the capacity to reduce metallic ions and stabilize the synthesized NPs. The functional groups in the plant phytoconstituents (dimethyl silanediol, catechol, diethyl phthalate, caffeine, l-(+)-ascorbic acid 2,6-dihexadecanoate, stigmasterol, cholesta-4,6-dien-3beta-ol and stigmastan-3,5-diene) were able to stabilize the NPs as noted by a highly negative zeta potential value (Figure 2). By capping the NPs, the magnitude of electrostatic attraction between the NPs can be reduced, thereby limiting NP aggregation and improving their dispersity. Further confirmation of the successful synthesis of Ag@Se NPs was the dominant signals of Ag (3.2 KeV) and Se (1.4 and 11.2 KeV) from EDX spectroscopy which corresponded with previous reports on the green synthesis of Ag and Se NPs [46,47].

The suitability of NPs for biological applications is largely dependent on their physiochemical properties. Thus, zeta potential, size, and polydispersity values of NPs must fall within prescribed limits. Although NPs smaller than 200 nm have the potential to traverse most biological barriers, those less than100 nm may be more likely to pass through barriers such as the blood-brain barrier [48]. In this study, the NP size from EM was below 50 nm, while that obtained from NTA was larger at 134 nm. This can be expected as NTA provides the size and charge of NPs in aqueous solutions compared to the dry samples used in EM and closely mimics what one may experience in an in vivo system in the presence of biological fluids. Hence, the hydrodynamic size obtained depended upon the hydration layer and NP corona [49]. It was reported that Ag_2_Se NPs of 20–40 nm in size had significant antioxidant, anticancer, and antibacterial properties [50]. In addition, varying degrees of photoluminescence of Ag_2_Se NPs with increasing sizes from 8–96 nm using different capping agents was observed [16]. The smaller NPs appeared to favor the photoluminescence properties in the NPs. Furthermore, Ag_2_Se NPs around 10 nm in size was observed to show photothermal properties, which could see their application in theranostics [51]. Generally, chalcogenides have been reported to have size-tunable photoemission properties within a size range of 1–20 nm [52]. Similarly, NPs between 20–50 nm have shown significant tumor penetration and increased cellular uptake [53,54]. NPs exceeding 200 nm in size have been shown to trigger the complement system and are easily cleared from the body [55]. Conversely, it has been noted that NPs of the order of 10 nm and below are easily cleared by the renal system [56]. Hence, the optimal size of a NP ultimately depends on the therapeutic application of the NP. Nonetheless, from a cancer therapeutic perspective, NP sizes ranging from 20–50 nm are highly desirable [57]. 

Among the factors that can alter the structure of NPs, are the amount of plant extract used in the reduction reaction and the reaction temperature. During the synthesis of Ag@Se BNPs using quercetin and gallic acid, it was reported that an increase in the precursor ion concentration from 0.5 to 1.0 mM significantly increased the number of NPs formed [15]. The reaction temperature was also reported to have a significant influence on the formation of Ag_2_Se NPs. This study evaluated the impact of the amount of extract and reaction temperature for the optimal synthesis of Ag@Se NPs with desirable stability and dimension. However, contrary to previous experiments based on changing a single variable at a time, statistical optimization was employed in the current study. Based on the central composite design, the independent variables were fitted into a second-order polynomial, which uses the least square technique. The analysis observed that the NPs’ stability, size, and yield could be controlled by altering the amount of extract and temperature. Highly stable NP synthesis was observed at high temperatures together with a high extract volume. Optimal synthesis of NPs was achieved at 110 °C and with an extract volume within the range of 500–1700 μL with the desirability function value close to 1. The increase in NP yield at high temperatures could result from an increase in the rate of the reaction that was accompanied by an increased collision of reactant particles. In addition, increasing the amount of the extract could improve the yield by providing more reducing agents required for the complete reduction of the metallic ions. The analysis further demonstrated that temperature appeared to be a significant factor in controlling the size of the NPs, as observed by the *p*-value.

Contrary to findings in a previous study that reported that a decrease in temperature favored the synthesis of small-sized Ag-Se BNPs [51], our result showed that high temperature appeared to favor the synthesis of smaller NPs. This might be due to the slow reduction of metallic ions at low temperatures leading to decreased number of nucleation centers [58,59]. Minimal nucleation sites might result in rapid aging and enlargement of NPs during the growth phase. Likewise, it was reported that increasing the reaction temperature from 130 °C to 190 °C significantly increased Ag_2_Se NP size [45]. Increased NP size with progressively higher temperature can occur due to rapid Ostwald ripening causing NP aging and enlargement [60]. The contrasting role of temperature in NP size is still a subject of research, but in this experiment, a low temperature would have significantly altered the Ag@Se NPs yield. Hence, high temperature appears to be more crucial for the green synthesis of Ag@Se NPs.

Apart from the benefits mentioned earlier, the reason for synthesizing NPs through the green route is the interesting biological activities imbued in biogenic NPs. The Ag@Se NPs synthesized in this study demonstrated free radical scavenging activity, a property linked to the presence of the phenolics and flavonoids from the plant extract that served as capping agents of the NPs. Free radicals have been implicated in the pathophysiology of many diseases such as cancer, cardiovascular diseases, diabetes, inflammation, and neurodegeneration [61]. By scavenging free radicals, the Ag@Se NPs can be utilized in preventing oxidative damage. While it is noted that the NPs demonstrated similar antioxidant activity to ascorbic acid, they showed a much lower antioxidant activity compared to the plant extract. This may be due to the extract containing additional compounds with antioxidant activities that were either absent or possibly present in insignificant amounts in the NPs.

Further emphasizing the biological potential of Ag@Se NPs is the observed cytotoxic effect in the breast cancer cells at high concentration and their biocompatibility at a lower concentration. The cytotoxicity can be due to the presence of Ag and Se or the capping agent [15,29,50]. It was recently reported that AgNPs synthesized by citrate reduction produced similar high cytotoxicities in MCF-7 cells at higher concentrations [62]. It should be noted that, unlike AgNPs, Ag@Se NPs appear to be less cytotoxic [29]. Furthermore, SeNPs due to their desirable properties, have been investigated in therapeutic gene and drug delivery, and it has been proposed that they may work synergistically with drug or gene being transported [63]. Hence, the addition of Se, may have reduced some of the inherent toxicity of the Ag within the BNPs. Moreover, the synthesis of Ag@Se NPs through green synthesis improved the NPs biocompatibility [50], therefore enhancing their suitability as drug and gene delivery vehicles in efforts to formulate more effective modalities to combat cancer [15,29,64].

NPs have been shown in different studies to pose a threat not just to humans but also to plants. There is currently no data available with regards to the toxicity of Ag@Se NPs in any plant species. In the current study, we further demonstrated that Ag@Se NPs inhibited the growth of *Allium cepa* root cells in a dose-dependent manner at elevated concentration levels and may induce genotoxic damage. The NPs elicit a mitodepressive effect, interfering with normal cell development by preventing quiescent cells (cells in interphase) from progressing through the cell cycle. The uptake of NPs by the plant root cells was shown to induce stress responses, causing the activation of cellular organelles as a defensive mechanism to prevent stress-induced cellular damage in the plant tissue. Cellular stress induction at the high concentration by NPs might involve reactive oxygen species (ROS) production and result in cell death. It can be suggested that when Ag@Se NPs come in contact with the *Allium cepa* root, due to their nanometric size, they may be adsorbed on the root surface and slowly diffuse through the pores of the cell wall into the cytoplasm. Following cytoplasmic infiltration, they can be sequestered within the vacuoles or attach to mitochondria causing mitochondria permeabilization. The loss of mitochondrial potential leads to the release of ROS from the electron transport chain leading to cellular damage. Overall, the results from an *Allium cepa* assay provides a good correlation with multicellular test systems that utilize mouse or rabbits models, validating its use for genotoxic studies [36].

Computational analysis of the identified constituents from the plant extract suggested that the capping agent did not pose genotoxic stress and were not capable of forming strong interactions with the DNA, which would stimulate DNA damage. This observation suggested that rather than rendering the NPs genotoxic, the use of plant-based material to synthesize Ag@Se NPs possibly acted to improve their compatibility [50]. It has been reported that *Ocimum sanctum* extract has been used in the synthesis of AgNPs that have been employed as genoprotective agents [65]. In the present study, we have demonstrated that *Ocimum tenuiflorum* extract was effective in the synthesis of biocompatible Ag@Se NPs.

## 5. Conclusions

The demand for NPs with desireable biological properties, that are safe and inexpensive, has spurred research into the use of plant extracts in green syntheses approaches. *Ocimum tenuiflorum* is one of the most commonly reported plants used to synthesize NPs due to its profound antioxidant potential that allows it to easily reduce metallic ions to generate NPs. The current study is the first report on the successful use of *Ocimum tenuiflorum* to synthesize Ag@Se BNPs. The electron-donating capacity of *Ocimum tenuiflorum* was explored to reduce Ag and Se ions to form Ag@Se NPs successively. Response surface methodology explained the effect of the amount of extract and reaction temperature on NP structure. It ensured the optimal synthesis of Ag@Se NPs with the desired particle size and stability. The NPs synthesized demonstrated significant free radical scavenging activity and a dose-dependent cytotoxic and genotoxic effect. At concentrations below 60 µg/mL, these NPs showed very favorable biocompatibility that can be explored further in therapeutic drug and gene delivery. However, at high concentrations, these NPs can pose significant threats to both cancer and normal cell lines and are capable of causing genotoxic damage.

## Figures and Tables

**Table 1:** Gas chromatography-mass spectrometry (GC-MS)-based tentative identification of components in aqueous extracts of *Ocimum tenuiflorum* inflorescence.

Retention Time	Peak Area (mm^2^)	Peak Area (%)	Peak Height (mm)	Peak Height (%)	A/H	Molecular Formula	Tentative Compound	NIST LIB Database (CAS) ID	NIST Similarity Index (%)
4.225	152.678	1.13	127.942	4.02	1.19	C_2_H_8_O_2_Si	Dimethyl silanediol	1066-42-8	95
4.775	76.412	0.57	16.309	0.51	4.69	C_6_H_6_O_2_	Catechol	120-80-9	72
9.232	103.742	4 7.69	149.341	4.69	6.95	C_12_H_14_O_4_	Diethyl Phthalate	84-66-2	97
16.049	1.831.956	13.58	316.649	9.94	5.79	C_8_H_10_N_4_O_2_	Caffeine	58-08-2	98
17.049	356.096	2.64	85.134	2.67	4.18	C_38_H_68_O_8_	l-(+)-Ascorbic acid 2,6-dihexadecanoate	28474-90-0	90
21.403	880.775	6.53	100.591	3.16	8.76	C_29_H_48_O	Stigmasterol	83-48-7	90
22.837	134.922	1.00	19.079	0.60	7.07	C_27_H_44_O	Cholesta-4,6-dien-3beta-ol	14214-69-8	60
23.290	253.161	1.88	46.322	1.45	5.47	C_29_H_48_	Stigmastan-3,5-diene	0-00-0	65

**Table 2:** Central composite design-based experimental runs and response variables for the synthesis of Ag@Se NPs.

Sample	Extract (µL)	Temperature (°C)	Zeta Potential (mV)	Hydrodynamic Size (nm)	Concentration Particles/mL (×10^10^)
1	500.0	100.00	−22.8	48.0	6.090
2	1500.0	50.00	−17.8	77.4	0.249
3	1000.0	39.65	−24.6	83.9	0.296
4	1000.0	75.00	−20.1	62.4	4.095
5	1000.0	75.00	−20.8	55.9	4.100
6	1000.0	75.00	−24.0	59.5	3.950
7	292.9	75.00	−25.2	62.7	0.354
8	1000.0	75.00	−26.9	58.8	3.900
9	1500.0	100.0	−30.4	64.1	8.550
10	1707.1	75.00	−28.4	42.1	4.380
11	500.0	50.00	−29.4	55.2	0.584
12	1000.0	110.36	−28.6	38.0	7.950
13	1000.0	75.00	−24.0	55.9	4.250

**Table 3:** Optimal condition prediction based on response surface model.

Parameter	Response Variable	Extract (µL)	Temperature (°C)	Response Value	Composite Desirability
1	Zeta potential fit	1707.110	110.355	−40.394 mV	1.000
2	Hydrodynamic size	507.168	110.355	44.908 nm	0.849
3	Concentration	1707.110	110.355	1.019 × 10^11^ particles/mL	1.000

**Table 4:** The IC_50_ value for antioxidant and cytotoxicity studies on Ag@Se NPs.

Nanoparticle/Drug	DPPH (µg/mL)	FRAP (µg/mL)	HEK293 (µg/mL)	MCF-7 (µg/mL)
Ag@Se	13966.93	11.02	1649.65	456.38
Ascorbic acid	205.64	15.66	-	-
5FU	-	-	111.29	136.71
Extract	112.34	10.98	-	-

**Table 5:** Mitotic division rate per 1000 cells scored in control and Ag@Se NP treated *Allium cepa* root cells.

Treatment	Sample	I ^a^ (%)	P ^b^ (%)	M ^c^ (%)	A ^d^ (%)	T ^e^ (%)	MI ^f^ (%)	Ave MI (%)	MD ^g^ (%)	Ave MD (%)
Distilled H_2_O	Sample 1	61.49	32.76	1.72	1.15	2.87	38.51	38.03 ± 1.44	−1.25	0.00 ± 3.79
	Sample 2	60.82	25.26	6.70	2.06	5.15	39.18		−3.01	
	Sample 3	63.59	26.15	7.69	1.54	1.03	36.41		4.26	
Ag@Se (1 µg/mL)	Sample 1	68.26	27.54	1.80	1.20	1.20	31.74	33.77 ± 4.26	16.55	11.19 ± 11.19
	Sample 2	69.08	27.63	1.97	0.66	0.66	30.92		18.69	
	Sample 3	61.33	34.67	0	2.67	1.33	38.67		−1.67	
Ag@Se (10 µg/mL)	Sample 1	77.33	20.89	0.44	0.44	0.89	22.67	28.19 ± 5.53	40.40	25.87 ± 14.53
	Sample 2	71.81	22.55	1.48	2.97	1.19	28.19		25.87	
	Sample 3	66.28	31.39	1.16	0.58	0.58	33.72		11.33	
Ag@Se (100 µg/mL)	Sample 1	69.85	25.19	3.44	0.76	0.76	30.15	28.03 ± 3.43	20.71	26.30 ± 9.01
	Sample 2	75.93	17.28	1.23	3.70	1.85	24.07		36.70	
	Sample 3	70.14	27.49	0.95	0.47	0.95	29.86		21.49	

**Table 6:** Cytological effects per 1000 cells scored in the control and Ag@Se NP treated *Allium cepa* root cells.

Treatment	Sample	Vagrant	Binuclei	Sticky	C-metaphase	Laggard	Chromosomal Aberration	CAI * (%)	Average CAI
Distilled H_2_O	Sample 1	0	1	0	0	0	1	0.1	0.13 ± 0.06
	Sample 2	0	1	0	0	0	1	0.1	
	Sample 3	0	2	0	0	0	2	0.2	
Ag@Se (1 µg/mL)	Sample 1	1	1	2	0	1	5	0.5	0.60 ± 0.10
	Sample 2	1	3	1	0	2	7	0.7	
	Sample 3	2	2	0	1	1	6	0.6	
Ag@Se (10 µg/mL)	Sample 1	2	1	0	1	2	6	0.6	0.63 ± 0.06
	Sample 2	1	2	1	2	1	7	0.7	
	Sample 3	2	1	0	1	2	6	0.6	
Ag@Se (100 µg/mL)	Sample 1	3	6	5	1	4	19	1.9	1.67 ± 0.25
	Sample 2	2	5	2	3	2	14	1.4	
	Sample 3	3	3	5	2	4	17	1.7	

CAI * = chromosomal aberration index.

**Table 7:** Molecular modeling and interaction profiles of identified compounds and DNA.

Ligand	Standard Precision Docking Score	XP Docking Score	Interacting Residues
Dimethyl silanediol	−4.624	−4.224	DA (B: 17), DC (A: 9)
Catechol	−4.900	−4.093	DG (A: 10)
Diethyl Phthalate	−5.201	−1.928	DG (A: 10), DG (B: 16)
Caffeine	−6.282	−2.065	-
l-(+)-Ascorbic acid 2,6-dihexadecanoate	−1.972	4.987	DC (A: 9)
Stigmasterol	−2.339	−0.663	DG (A: 12)
Cholesta-4,6-dien-3beta-ol	−3.918	1.045	DG (A: 10)
Stigmastan-3,5-diene	−2.683	1.704	-
Quinacrine (standard)	−8.130	−8.983	DA (B: 17)

A = A-strand, B = B-strand, DA = deoxyadenosine, DG = deoxyguanosine, DC = deoxycytidine.

## Data Availability

The data and contributions presented in the study are included in the article and supplementary file. Further inquiries can be directed to the corresponding authors.

## Supplementary Materials

The following are available online at https://www.mdpi.com/article/10.3390/nano11102516/s1, Figure S1: GCMS chromatogram of phytoconstituents of *Ocimum tenuiflorum* inflorescence aqueous extract, Figure S2: 2-Dimensional structure of tentatively identified compounds from *Ocimum tenuiflorum* inflorescence aqueous extract, Figure S3: Mean root length of *Allium cepa* exposed to Ag@Se nanoparticles for 48 h, Figure S4: 3-Dimensional ligand interaction diagram of dimethyl silanediol (a), catechol (b), diethyl phthalate (c), caffeine (d), l-(+)-ascorbic acid 2,6-dihexadecanoate (e), stigmasterol (f), cholesta-4,6-dien-3beta-ol (g), stigmastan-3,5-diene (h), and quinacrine (i) with B-DNA. Table S1. Analysis of variance values and model parameters showing the effect of the amount of extract and temper-ature on zeta potential, hydrodynamic size and nanoparticle concentration.
