# Supplementary material for: Biogenic Synthesis of Silver-Core Selenium-Shell Nanoparticles Using Ocimum tenuiflorum L.: Response Surface Methodology-Based Optimization and Biological Activity
Source: Nanomaterials (Basel). 2021 Sep 27;11(10):2516. doi: 10.3390/nano11102516 (PMC8539562; doi:10.3390/nano11102516)
Supplement: Supplementary file 1 [file nanomaterials-11-02516-s001.zip › nanomaterials-1370619-supplementary.pdf]

nanomaterials

MDPI

Supplementary Material

Biogenic Synthesis of Silver-Core Selenium-Shell Nanoparticles Using *Ocimum tenuiflorum* L.: Response Surface Methodology-Based Optimization and Biological Activity

Femi Olawale, Mario Ariatti and Moganavelli Singh *

Nano-Gene and Drug Delivery Group, Discipline of Biochemistry, School of Life Sciences, College of Agriculture, Engineering and Science, University of KwaZulu-Natal, Private Bag X54001, Durban, 4000, South Africa; 219094535@stu.ukzn.ac.za (F.O.); ariattim@ukzn.ac.za (M.A.)

* Correspondence: singhm1@ukzn.ac.za; Tel.: +2731-2607170

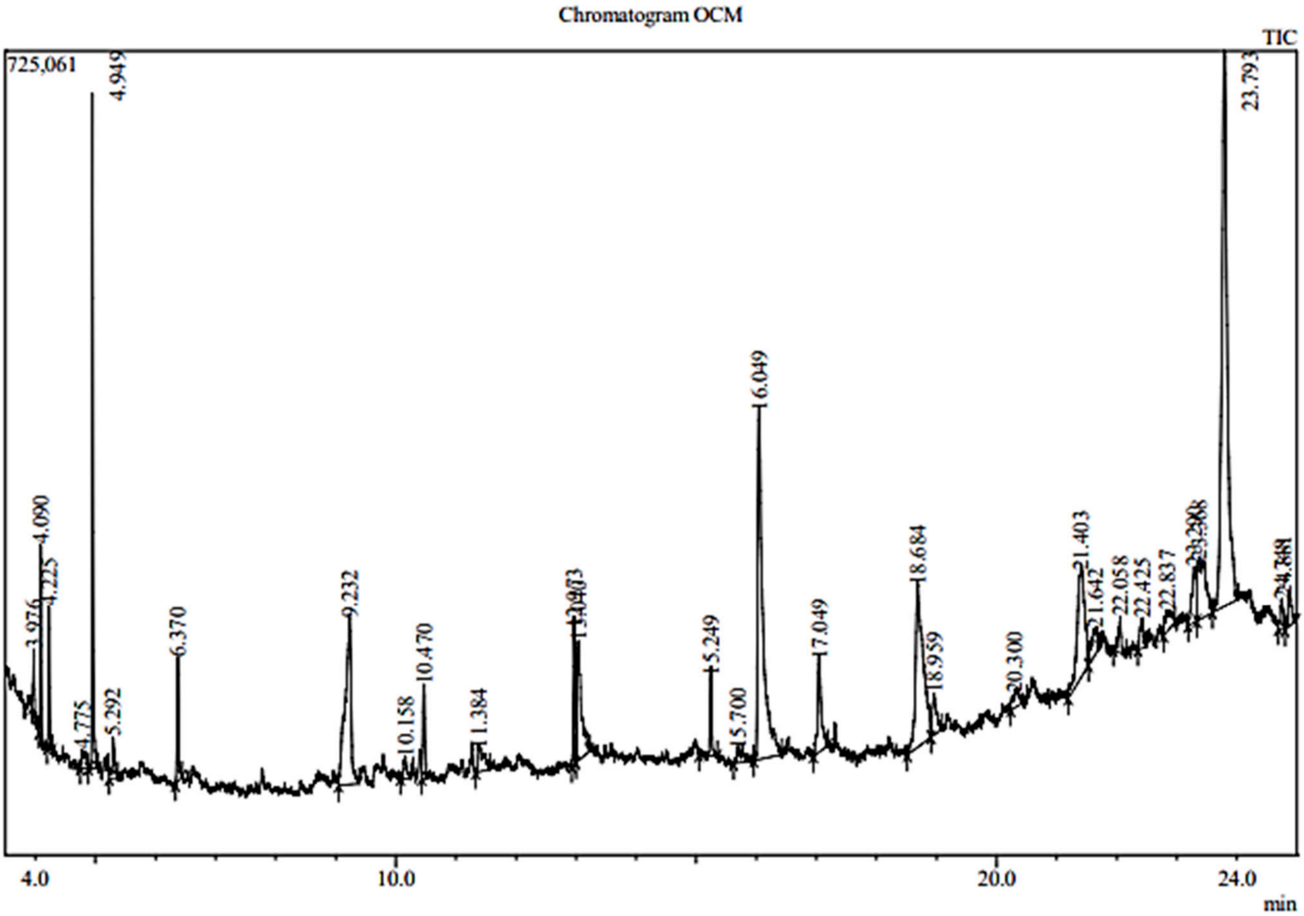

Figure S1. GCMS chromatogram of phytoconstituents of *Ocimum tenuiflorum* inflorescence aqueous extract.

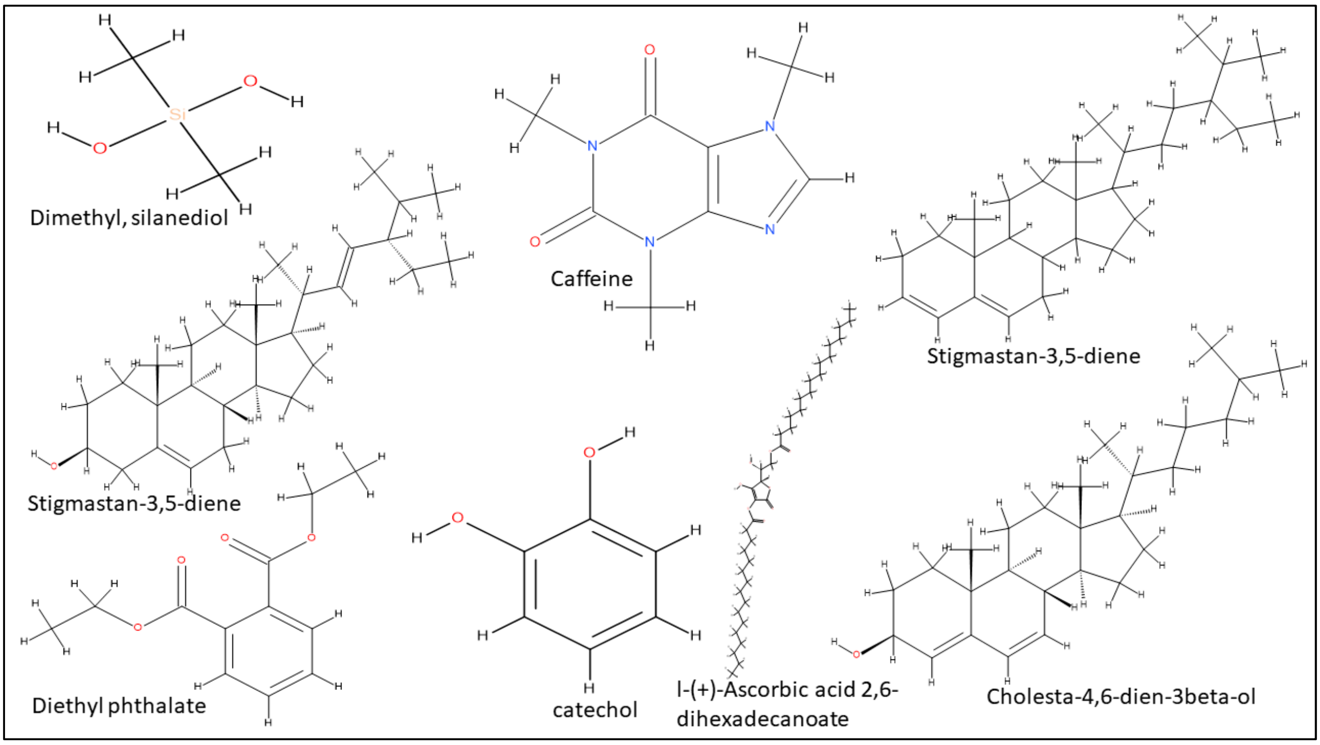

Figure S2. 2-Dimensional structure of tentatively identified compounds from *Ocimum tenuiflorum* inflorescence aqueous extract.

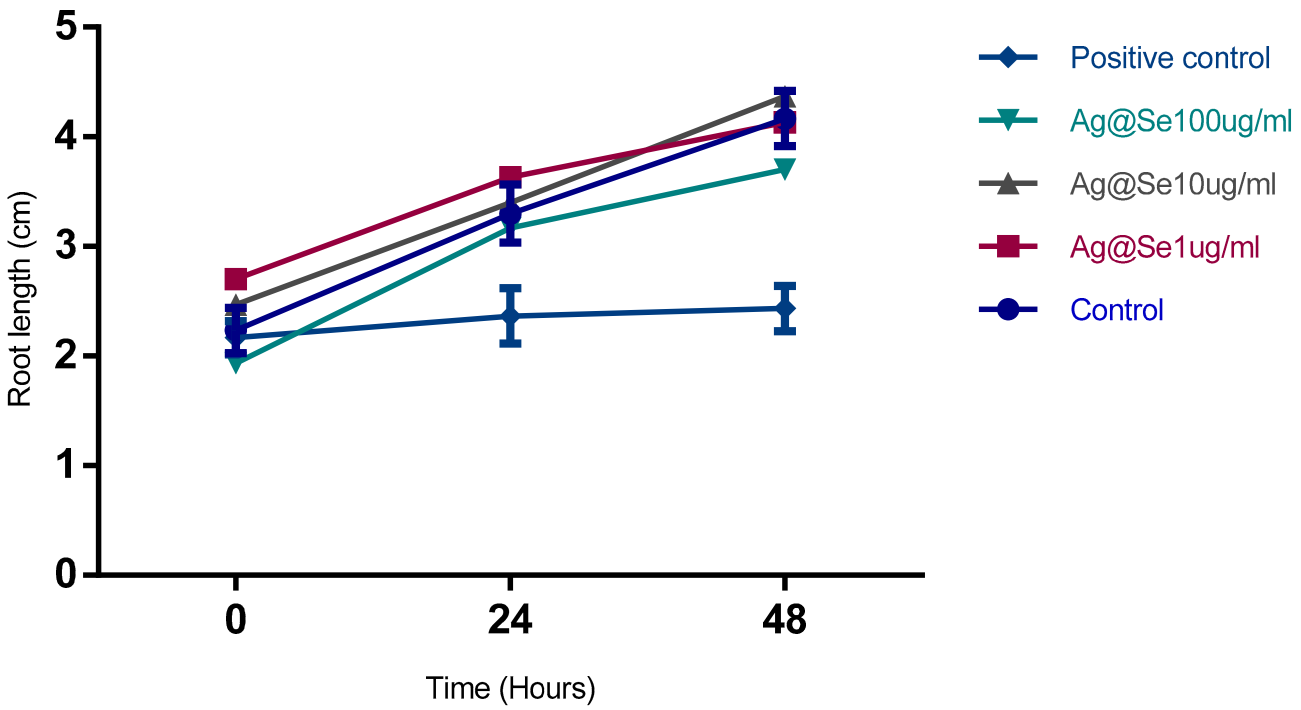

Figure S3. Mean root length of *Allium cepa* exposed to Ag@Se nanoparticles for 48 hours.

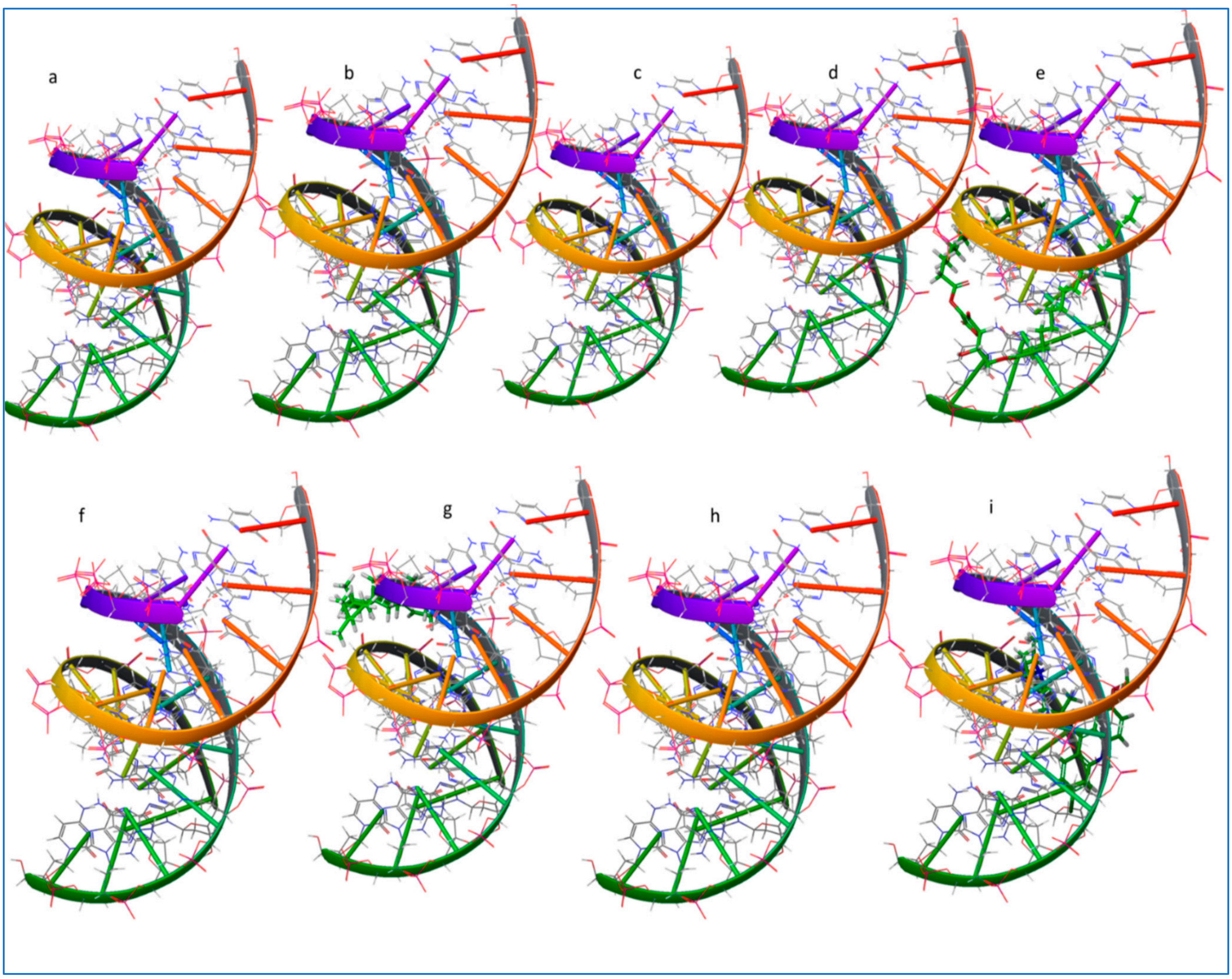

Figure S4. 3-Dimensional ligand interaction diagram of dimethyl silanediol (a), catechol (b), diethyl phthalate (c), caffeine (d), l-(+)-ascorbic acid 2,6-dihexadecanoate (e), stigmasterol (f), cholesta-4,6-dien-3beta-ol (g) stigmastan-3,5-diene (h) and quinacrine (i) with B-DNA.

Supplementary Table S1. Analysis of variance values and model parameters showing the effect of the amount of extract and temperature on zeta potential, hydrodynamic size and nanoparticle concentration.

**Zeta potential**					
**Source**	**DF**	**Adj SS**	**Adj MS**	**F-Value**	**P-Value**
Model	5	132.320	26.4640	4.16	0.045
Linear	2	17.020	8.5099	1.34	0.322
EXTRACT	1	0.035	0.0345	0.01	0.943
TEMPERATURE	1	16.985	16.9853	2.67	0.146
Square	2	23.140	11.5702	1.82	0.231
EXTRACT*EXTRACT	1	14.027	14.0271	2.20	0.181
TEMPERATURE*TEMPERATURE	1	12.121	12.1210	1.90	0.210
2-Way Interaction	1	92.160	92.1600	14.48	0.007
EXTRACT*TEMPERATURE	1	92.160	92.1600	14.48	0.007
Error	7	44.552	6.3646		
Lack-of-Fit	3	14.220	4.7401	0.63	0.636
Pure Error	4	30.332	7.5830		

Total	12	176.872			
Model summary	R^2 = 74.81% , R^2 (adj)= 56.82%				
**Hydrodynamic size**					
**Source**	**DF**	**Adj SS**	**Adj MS**	**F-Value**	**P-Value**
Model	5	1076.38	215.276	2.91	0.098
Linear	2	1054.50	527.250	7.12	0.021
EXTRACT	1	142.59	142.590	1.93	0.208
TEMPERATURE	1	911.91	911.910	12.32	0.010
Square	2	12.58	6.289	0.08	0.919
EXTRACT*EXTRACT	1	7.63	7.633	0.10	0.758
TEMPERATURE*TEMPERATURE	1	6.58	6.579	0.09	0.774
2-Way Interaction	1	9.30	9.303	0.13	0.733
EXTRACT*TEMPERATURE	1	9.30	9.303	0.13	0.733
Error	7	518.20	74.029		
Lack-of-Fit	3	475.86	158.618	14.98	0.012
Pure Error	4	42.35	10.587		
Total	12	1594.58			
Model summary	R^2 = 67.50% , R^2 (adj)= 44.29%				
**Concentration**					
**Source**	**DF**	**Adj SS**	**Adj MS**	**F-Value**	**P-Value**
Model	5	89.2926	17.8585	35.14	0.000
Linear	2	83.4795	41.7398	82.13	0.000
EXTRACT	1	7.6414	7.6414	15.04	0.006
TEMPERATURE	1	75.8382	75.8382	149.23	0.000
Square	2	3.8601	1.9300	3.80	0.076
EXTRACT*EXTRACT	1	3.3138	3.3138	6.52	0.038
TEMPERATURE*TEMPERATURE	1	0.2454	0.2454	0.48	0.510
2-Way Interaction	1	1.9530	1.9530	3.84	0.091
EXTRACT*TEMPERATURE	1	1.9530	1.9530	3.84	0.091
Error	7	3.5574	0.5082		
Lack-of-Fit	3	3.4808	1.1603	60.57	0.001
Pure Error	4	0.0766	0.0192		
Total	12	92.8500			
Model summary	R^2 = 96.17% , R^2 (adj)= 93.43%				
